# Fear of Missing Organisms (FOMO): the discordance among broad-spectrum empiric antibiotic therapy, microbiologic results, and definitive antibiotic therapy for diabetic foot infections and lower extremity osteomyelitis

**DOI:** 10.1017/ash.2023.467

**Published:** 2023-10-25

**Authors:** Morgan K. Morelli, Andrea H. Son, Yanis Bitar, Michelle T. Hecker

**Affiliations:** 1 Department of Medicine, Division of Infectious Diseases and HIV Medicine, University Hospitals Cleveland Medical Center, Case Western Reserve University, Cleveland, OH, USA; 2 Department of Medicine, Division of Infectious Disease, The MetroHealth System, Case Western Reserve University, Cleveland, OH, USA; 3 Department of Pharmacy, The MetroHealth System, Case Western Reserve University, Cleveland, OH, USA

## Abstract

**Objective:**

Empiric broad-spectrum antibiotic therapy is commonly prescribed for patients hospitalized with diabetic foot infections (DFI) and lower extremity osteomyelitis (OM). The primary objective was to evaluate the concordance between empiric antibiotic therapy, microbiologic results, and definitive antibiotic therapy with a focus on methicillin-resistant *Staphylococcus aureus* (MRSA) and resistant gram-negative organisms. The secondary objective was to evaluate the negative predictive values (NPV) of select risk factors for MRSA and resistant gram-negative organisms for microbiologic results with these organisms.

**Design:**

Retrospective cohort study.

**Setting:**

Safety-net health system in Ohio.

**Patients:**

Adults hospitalized and receiving antibiotic therapy for DFI or lower extremity OM in 2021.

**Results:**

For 259 unique patients, empiric therapies with activity against MRSA and resistant gram-negative organisms were administered to 224 (86.5%) and 217 (83.8%) patients, respectively. Definitive therapies with activity against MRSA and resistant gram-negative organisms were administered to 91 (35%) and 74 (28.6%) patients, respectively. Of 234 patients with microbiologic testing, 29 (12.4%) had positive cultures with MRSA and 41 (17.5%) with resistant gram-negative organisms. The NPVs of risk factors for MRSA and resistant gram-negative organisms for the absence of these organisms in culture were 91% and 85%, respectively.

**Conclusions:**

For patients hospitalized with DFI and lower extremity OM, our data suggest opportunities for substantial reductions in empiric therapies with activity against MRSA and resistant gram-negative organisms. The absence of risk factors for these organisms was reasonably good at predicting negative cultures with these organisms.

## Introduction

Hospitalizations for diabetic foot infections (DFI) and lower extremity osteomyelitis (OM) are common.^1,2^ Current guidelines recommend selection of empiric antibiotics based on severity of infection, risk factors for or history of colonization or infection with methicillin-resistant *Staphylococcus aureus* (MRSA) and *Pseudomonas aeruginosa*, and local prevalence of these organisms.^3,4^ Prior studies documented high rates of unnecessary empiric anti-MRSA and anti-pseudomonal therapy^5,6^ based on correlation with microbiologic results; however, most of these studies excluded patients with negative or no microbiologic testing. In “real-world” settings, microbiologic testing may not be performed, or testing may be negative due to inadequate sampling, sampling after the initiation of antibiotics, or absence of true infection. Many of these patients are nonetheless treated with both empiric and definitive antibiotic therapies. For these reasons, as well as because providers may deem some organisms isolated in culture as colonizers not requiring treatment, describing the concordance of empiric therapy and definitive therapy provides additional information about the “appropriateness” of the empiric therapy. Expanding the evaluation to other resistant gram-negative organisms, in addition to *P. aeruginosa*, is also important given recent studies documenting an unexpectedly high prevalence of positive cultures with these organisms.^7^ Empiric anti-pseudomonal therapy may be appropriate when including these resistant gram-negative organisms. Finally, evaluation of the negative predictive values (NPV) of commonly used risk factors for MRSA and resistant gram-negative organisms, including *P. aeruginosa*, to inform decision-making about avoiding broad-spectrum empiric antibiotic therapy in patients hospitalized with these diagnoses would be useful.

The primary objective of this quality improvement project was to evaluate the concordance between empiric antibiotic therapy, microbiologic results, and definitive antibiotic therapy with a focus on MRSA and resistant gram-negative organisms. The secondary objective was to evaluate the NPV of select risk factors, adapted from those described in the 2019 Infectious Diseases Society of America community-acquired pneumonia guidelines,^8^ for MRSA and resistant gram-negative organisms for microbiologic results with these organisms.

## Methods

A retrospective cohort study of patients hospitalized with DFI and/or lower extremity OM between January and December 2021 was conducted at three hospitals in a safety-net medical system in Northeast Ohio. Patient admissions with associated ICD-10 diagnosis codes M86, E10.621, E11.621, E08.621, A48.0, or I96 were included. Patient admissions were excluded if the antibiotics given during the hospitalization were for another indication, if a hardware infection was suspected without the presence of diabetes or peripheral artery disease (PAD), or if patients were younger than 18 years old. For patients with multiple hospitalizations during 2021, only the first hospitalization for this indication was included.

Data collected included demographics, clinical status, microbiology results, radiology results, antibiotic therapy, consultations, operative reports, and select clinical outcomes. Each case was classified based on the International Working Group on the Diabetic Foot (IWGDF) guideline classification system (Table 1).^4,9^ Wound classifications were based on the characteristics of the wound described in the inpatient admission note and podiatry consult note, when available. Empiric antibiotic therapy was defined as the antibiotics given by the inpatient team during the first 72 h of admission. Definitive antibiotic therapy was defined as the final choice of antibiotics (after the first 72 h of admission), either completed during admission or prescribed at the time of discharge. Anti-MRSA antibiotics included vancomycin, daptomycin, linezolid, trimethoprim-sulfamethoxazole, doxycycline, clindamycin, dalbavancin, ceftaroline, and eravacycline. Antibiotics to treat resistant gram-negative organisms included piperacillin-tazobactam, cefepime, meropenem, ciprofloxacin, levofloxacin, aztreonam, and ceftazidime. Ertapenem was not classified as an antibiotic to treat resistant gram-negative organisms in the context of this study.

**Table 1.** Classification of foot infections based on the International Working Group of the Diabetic Foot criteria

IWGDF classification	Clinical classification
Infection	Presence of at least 2: - Local swelling - Erythema >0.5 cm around the wound - Local tenderness or pain - Local increased warmth - Purulent discharge
2 (mild infection)	Infection without systemic manifestations and involving: - Only the skin or subcutaneous tissues - Erythema that does not extend >2 cm from the wound
3 (moderate infection)	Infection without systemic manifestations and involving: - Erythema extending ≥2 cm from the wound AND/OR - Tissue deeper than skin and subcutaneous tissues
4 (severe infection)	Any infection with associated systemic manifestations with at least 2: - Temperature >38° C or <36° C - Heart rate >90 beats per minute - Respiratory rate >20 breaths per minute - White blood cell count >12,000 mm^3^ or <4,000 mm^3^
Add (O) after 3 or 4*	Infection involving bone

Note. *Osteomyelitis is classified as either 3(O) (with <2 systemic manifestations) or 4(O) (with ≥2 systemic manifestations).

Microbiologic testing was performed at the provider’s discretion. None of the three hospitals routinely screened patients for MRSA on admission. Superficial cultures for which only “normal skin flora” were reported were considered to have “no significant growth.” Isolation of MRSA or resistant gram-negative organisms from any type of wound culture (superficial, debridement, or surgical) or from a blood culture obtained during the index admission or within 7 d before the index admission was considered a positive microbiologic result. Resistant gram-negative organisms included *P. aeruginosa*, *Enterobacterales* with intrinsic resistance to ampicillin-sulbactam including *Citrobacter freundii*, *Enterobacter cloacae*, *Klebsiella aerogenes*, *Serratia marcescens*, and *Hafnia* spp.,^10^ and gram-negative bacilli with documented acquired resistance to both ceftriaxone and ampicillin-sulbactam. MRSA risk factors included prior culture (at any site, including MRSA nares cultures) from which MRSA was isolated within the last year, hospitalization with intravenous (IV) antibiotics within 90 d, IV drug use, or chronic hemodialysis. Risk factors for resistant gram-negative organisms included prior culture (at any site) from which a resistant gram-negative organism was isolated within the last year or hospitalization with IV antibiotics within 90 d.

Data were collected as counts and percentages using Microsoft Excel. Statistical analysis was performed using SAS 9.4. The agreement between empiric and definitive therapies was measured using the McNemar test. A chi-squared test was used to evaluate the concordance between microbiologic results and empiric and definitive therapies. Positive predictive values (PPV) and NPV were used to assess the risk factor’s ability to predict microbiologic results with MRSA and resistant gram-negative organisms. This was approved as a quality improvement project.

## Results

Of 533 adult patients hospitalized in 2021 with DFI and lower extremity OM-related diagnosis codes, 274 were excluded: 192 due to infection not being in the lower extremity, 63 due to the use of antibiotics for another indication, and 19 due to a suspected hardware infection without the presence of diabetes or PAD. Of the 259 patients included in the study, 72 had more than one admission for this indication during the study period. Baseline characteristics of the patients, including classification of their wounds, are shown in Table 2. Diabetes was present in 86.1%, PAD in 68.3%, and 2.3% had neither diabetes nor PAD, including 4 patients with neuropathy due to spinal cord injury and 2 patients with non-diabetic Charcot arthropathy. Osteomyelitis was diagnosed in 165 patients (63.7%). Twenty-six percent of cases were classified as severe [4 or 4(O)]. Sixty-two percent of cases were classified as moderate [3 or 3(O)]. During the index admission, 112 (43.2%) patients had an amputation (including 53 digit amputations, 26 forefoot amputations, and 33 above/below knee amputations), 48 (16.6%) had surgical debridement, and 92 (35.5%) had bedside debridement.

**Table 2.** Baseline characteristics

Number of patients	259
Average age (years)	61.1
**Sex**	
Male	181 (69.9)
Female	78 (30.1)
**Race**	
White	163 (62.9)
Black	90 (34.7)
Other	6 (2.3)
**Comorbidities**	
Diabetes	223 (86.1)
Average hemoglobin A1c	8.8%
Peripheral arterial disease	177 (68.3)
Hardware	9 (3.5)
Intravenous drug use	7 (2.7)
End-stage renal disease on dialysis	15 (5.8)
**IWGDF Classification**	
2	31 (12)
3	47 (18.1)
4	17 (6.6)
3(O)	114 (44)
4(O)	50 (19.3)
**Consultants**	
Infectious disease	177 (68.3)
Podiatry	226 (87.3)
Vascular surgery	114 (44)
**Lower extremity signs** (any of below)	111 (42.9)
Purulence	77 (29.7)
Gas on X-ray	17 (6.6)
Necrosis/abscess/tenosynovitis/septic arthritis on imaging	35 (13.5)
**MRSA risk factors** (any)	95 (36.7)
Culture with MRSA in the past year	18 (6.9)
Hospitalization within last 90 days with IV antibiotic use	73 (28.2)
Intravenous drug use	7 (2.7)
Chronic hemodialysis	15 (5.8)
**Resistant GNR risk factors** (any)	88 (34)
Culture with a resistant GNR in the past year	23 (8.9)
Hospitalization within last 90 days with IV antibiotic use	73 (28.2)

Note. GNR, gram-negative rod. IWGDF, International Working Group on the Diabetic Foot. MRSA, methicillin-resistant *Staphylococcus aureus*.

Two hundred thirty-four (90.3%) patients had at least one culture collected during their hospitalization or within 7 d before the index admission (Table 3). Overall, 79 (30.5%) patients had either no microbiologic testing (25, 9.7%) or negative microbiologic testing (54, 20.8%). MRSA was isolated in one or more cultures for 29 (12.4%) patients in whom any culture (including blood cultures) was obtained (11.2% of all patients). *P. aeruginosa* was isolated in one or more cultures for 17 (7.3%) patients in whom any culture was obtained (6.6% of all patients). An additional 26 (11.1%) patients had a non-pseudomonal resistant gram-negative organism isolated in one or more cultures (10% of all patients). Overall, 41 (17.5%) patients had at least one resistant gram-negative organism, including *P. aeruginosa*, isolated in one or more cultures (15.8% of all patients).

**Table 3.** Microbiologic results

Organism	Any culture^a^	Any wound culture (debridement, surgical, superficial)	Debridement culture	Surgical culture	Superficial culture	Blood culture
	*N* = 234	*N* = 185^b^	*N* = 57	*N* = 99	*N* = 111	*N* = 161^c^
No significant growth^d^	140 (59.8)	33 (17.9)	6 (10.5)	16 (16.2)	14 (12.6)	127 (78.9)
*Staphylococcus aureus*	84 (35.9)	81 (43.8)	26 (45.6)	33 (33.3)	45 (40.5)	6 (3.7)
MRSA	29 (12.4)	28 (15.1)	9 (15.8)	11 (11.1)	18 (16.2)	2 (1.2)
MSSA	55 (23.5)	53 (28.6)	17 (29.8)	23 (23.2)	27 (24.3)	4 (2.5)
*Pseudomonas aeruginosa*	17 (7.3)	17 (9.1)	2 (3.5)	1 (1)	14 (12.6)	1 (0.6)
Non-pseudomonal resistant gram-negative^e^	26 (11.1)	25 (13.5)	3 (5.3)	10 (10.1)	15 (13.5)	2 (1.2)
Other susceptible gram negatives	54 (23.1)	53 (28.6)	12 (21.1)	16 (16.2)	40 (36)	6 (3.7)
*Streptococcus spp.*	60 (25.6)	53 (28.6)	18 (31.6)	16 (16.2)	30 (27)	10 (6.2)
*S. pyogenes*	3 (1.3)	3 (1.6)	2 (3.5)	0 (0)	1 (0.9)	0 (0)
*S. agalactiae*	35 (15)	35 (19)	14 (24.6)	11 (11.1)	18 (16.2)	3 (1.9)
Other	16 (6.8)	15 (8.1)	3 (5.3)	5 (5)	11 (9.9)	7 (4.3)
Coagulase-negative *staphylococci*	8 (3.4)	8 (4.3)	1 (1.8)	7 (7)	n/a	n/a
“Normal skin flora”	61 (26.1)	61 (33)	29 (50.9)	37 (37.3)	n/a	n/a
*Enterococcus spp.*	14 (6)	14 (7.6)	1 (1.8)	9 (9)	5 (4.5)	0 (0)
Anaerobes	43 (18.4)	42 (22.7)	8 (14)	20 (20.2)	14 (12.6)	4 (2.5)

Note. Patients could have more than one organism isolated in culture. Patients could have more than one type of wound culture.

a Any culture includes blood or any wound culture done during the admission or within 7 d of admission. Fifteen patients had a culture within 7 d of admission. Ten of these 15 also had cultures done during the admission.

b 54 (29.2%) has only superficial cultures collected.

c 47 (18.1%) had blood cultures without any wound cultures.

d Including “normal skin flora” reported as the only organisms present from superficial cultures; in 54 patients, all microbiologic testing was negative.

e *Enterobacter cloacae* (13), *Stenotrophomonas maltophilia* (5), *Serratia marcescens* (2), *Klebsiella pneumoniae* (2), *Alcaligenes faecalis* (2), *Proteus mirabilis* (2), *Citrobacter freundii* (1), “lactose non-fermenter” (1).

Twenty-nine (11.2%) patients had cultures obtained from the same site in subsequent encounters within 30 days after the index admission. Eight (3.1%) patients had either MRSA (3), *P. aeruginosa* (1), or non-pseudomonal resistant gram-negative organisms (4) isolated from those cultures which were not isolated in cultures from the index admission, of whom two had no microbiologic testing and two had negative microbiologic testing during the index admission. Six of these eight patients did not receive definitive therapy to treat these organisms during the index admission including two, one, and three patients with subsequent isolation of MRSA, *P. aeruginosa*, and non-pseudomonal resistant gram-negative organisms, respectively. Three of these patients had their antibiotics changed to target the new organisms, and one underwent an amputation within 30 days of the index admission.

**Figure 1.**
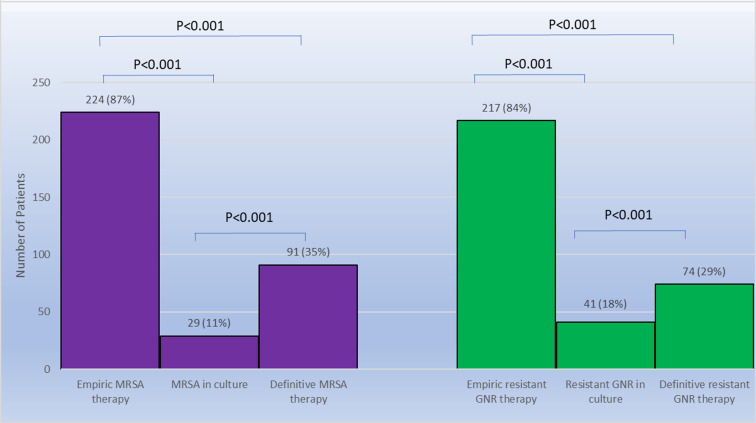
Concordance among empiric antibiotic therapy, culture results, and definitive antibiotic therapy for all patients. MRSA, methicillin-resistant *Staphylococcus aureus.*

Empiric therapies with activity against MRSA and resistant gram-negative organisms were administered to 224 (86.5%) and 217 (83.8%) patients, respectively. Definitive therapies with activity against MRSA and resistant gram-negative organisms were administered to 91 (35%) and 74 (28.6%) patients, respectively. Three patients in whom MRSA was isolated in culture were not given definitive therapy with activity against MRSA including two who had presumed source control with surgery. Ten patients in whom a resistant gram-negative organism was isolated in culture were not given definitive therapy with activity against resistant gram-negative organisms including five who had presumed source control with surgery. Although no patient received ertapenem as empiric therapy, 16 (6.2%) patients received ertapenem as definitive therapy, 13 of whom received it solely to simplify outpatient administration of parenteral antibiotic therapy and three of whom received it for treatment of resistant gram-negative organisms. The correlation between empiric therapies, microbiologic results, and definitive therapies is shown in Figure 1. There was a statistically significant difference between the antibiotic spectrum of empiric therapies administered and the microbiologic results and between the antibiotic spectrum of the empiric and definitive therapies for both MRSA and resistant gram-negative organisms (*p* < 0.0001). The data stratified by risk factors for MRSA and resistant gram-negative organisms are available in the supplementary table.

Table 4 shows the PPV and NPV of the risk factors for isolation of MRSA, *P. aeruginosa*, and all resistant gram-negative organisms (including *P. aeruginosa*). The NPV of MRSA risk factors was 91% for the absence of MRSA in culture. The NPV of *P. aeruginosa* risk factors was 95% for the absence of *P. aeruginosa* in culture. The NPV of risk factors for resistant gram-negative organisms was 85% for the absence of these organisms in culture.

**Table 4.** Predictive values for MRSA and resistant gram-negative organism risk factors with cultures for those organisms.

	PPV	NPV
MRSA	0.15 (95% CI 0.08–0.22)	0.91 (95% CI 0.87–0.96)
All resistant gram negatives	0.19 (95% CI 0.10–0.28)	0.85 (95% CI 0.80–0.91)
*Pseudomonas aeruginosa*	0.10 (95% CI 0.03–0.16)	0.95 (95% CI 0.92–0.98)

Note. CI, confidence interval. MRSA, methicillin-resistant *Staphylococcus aureus*. NPV, negative predictive value. PPV, positive predictive value.

## Discussion

In our healthcare system, greater than 80% of patients with DFI and lower extremity OM received empiric therapies with activity against MRSA and resistant gram-negative bacteria, but fewer than 20% of patients with microbiologic testing had positive cultures for these organisms. Fewer than 40% of the patients received definitive therapy with activity against MRSA or resistant gram-negative organisms. The NPV of commonly used risk factors for MRSA and resistant gram-negative organisms was greater than 85% for the absence of these organisms in microbiologic testing. These findings suggest opportunities for substantial reductions in empiric therapies with activity against MRSA and resistant gram-negative organisms for patients hospitalized with DFI and lower extremity OM.

Our findings are consistent with previous studies in the United States noting discrepancies between empiric anti-MRSA and anti-pseudomonal therapies and microbiologic results with these organisms.^6,11,12^ In contrast, in Western Australia, Hand *et al.*^13^ found a significant difference between the frequency of empiric anti-pseudomonal therapy and the frequency of isolation of *P. aeruginosa* in microbiologic testing; however, there was no significant difference between the frequency of empiric anti-MRSA therapy and the frequency of isolation of MRSA in microbiologic testing, 12.6% versus 11.9%, respectively. Notably, Western Australia has a well-established screening program for patients at high risk of MRSA with positive results prompting an electronic flag in the patient’s medical record.

Our findings build upon previous studies by evaluating discrepancies between anti-pseudomonal therapies and positive cultures for both *P. aeruginosa* and non-pseudomonal resistant gram-negative organisms. In a study of 648 patients, Henig *et al.*^7^ found *P. aeruginosa* in 94 (14.5%) patients during the index episode, ceftriaxone-resistant *Enterobacteriaceae* in 51 (7.9%), carbapenem-resistant *Enterobacteriaceae* in 6 (0.6%), and *Acinetobacter baumannii* in 22 (3.4%). In our study, 15.8% of patients had positive microbiologic tests for which anti-pseudomonal antibiotics or “antibiotics used to treat resistant gram-negative organisms,” as defined in our study, were appropriate. Notably, a greater number of patients had non-pseudomonal-resistant gram-negative organisms isolated in culture compared to those with only *P. aeruginosa* isolated in culture. This information is vital when developing recommendations for empiric therapy. Despite including these organisms, there remained a significant discrepancy between empiric anti-pseudomonal therapy and positive microbiologic results for both pseudomonal and non-pseudomonal resistant gram-negative organisms.

Our study also builds upon existing data by evaluating the discrepancy between empiric and definitive therapies. Although fewer than 10% of patients in our study had no microbiologic testing, nearly one-third had negative cultures. Yet, all these patients received antibiotic therapy. These patients would have been excluded from studies evaluating only patients with positive microbiologic testing. In addition, 13 (5%) patients had either MRSA or a resistant gram-negative organism isolated in culture but did not receive definitive therapy for these organisms either due to early definitive source control with no antibiotic therapy administered after 72 h of admission or due to providers considering these organisms to be colonizers/contaminants. Even with inclusion of these patients, a significant discrepancy remained between empiric and definitive therapies. In our study, positive cultures with MRSA or resistant gram-negative organisms within 30 d after the index admission were uncommon, including in patients for whom definitive therapy did not include this spectrum of activity, suggesting that we were unlikely to miss resistant organisms in patients who had either no microbiologic testing or negative microbiologic testing.

Several studies have identified risk factors independently associated with isolation of MRSA and/or resistant gram-negative organisms;^7,11,12,14^ however, these risk factors may have poor PPV and may lead to significant overuse of empiric broad-spectrum therapies. Our study focused on the NPV of risk factors with the goal of identifying patients for whom empiric broad-spectrum therapies could be withheld. Hand *et al*.^13^ found that the positive and negative predictive values of prior infection or colonization with MRSA for the subsequent recovery of MRSA were 54% and 97%, respectively. Mergenhagen *et al.*^5^ evaluated the utility of MRSA nares screening for patients with DFI. The NPV of MRSA nares screening for MRSA DFI was 89.6%. Our finding that a relatively simple set of commonly used risk factors for MRSA and resistant gram-negative organisms had a reasonably good NPV for ruling out positive cultures with these organisms adds to the current literature related to tools to help reduce unnecessarily broad empiric therapy.

Our study has several limitations. First, data were included from a single health system and might not be generalizable to other health systems. Second, a standardized microbiologic workup was not performed for all patients in this retrospective cohort. Third, positive culture results, especially from superficial cultures, may not accurately reflect the organisms “causing” the infection (false positive cultures). Alternatively, the culture results may have failed to identify the causative organisms (false negative cultures) due to inadequate sampling or sampling after the initiation of antibiotics. Fourth, for the few patients who had MRSA or resistant gram-negative organisms isolated from a culture within 30 d after the index admission which were not isolated during the index admission, it is not possible to determine whether this represented a new infection versus an organism that was present at the time of the index admission which was not identified during that admission. Finally, we assessed only a limited number of potential risk factors for MRSA and resistant gram-negative organisms. Including additional risk factors might have improved the NPV.

Despite these limitations, our data suggest an opportunity for significant reductions in empiric antibiotics targeting MRSA and resistant gram-negative organisms in DFI and lower extremity OM. Combining the risk factors for MRSA and resistant gram-negative organisms evaluated in this study with the severity of illness with or without the use of rapid molecular diagnostic tests should be evaluated as a strategy to optimize empiric antibiotic therapy in DFI and lower extremity OM.

## Supporting information

Morelli et al. supplementary material Morelli et al. supplementary material

## Supplementary material

For supplementary material accompanying this paper visit http://doi.org/10.1017/ash.2023.467. click here to view supplementary material

## Research transparency and reproducibility

A de-identified data set is available upon request.
