# Supplementary material for: Fear of Missing Organisms (FOMO): the discordance among broad-spectrum empiric antibiotic therapy, microbiologic results, and definitive antibiotic therapy for diabetic foot infections and lower extremity osteomyelitis
Source: Antimicrob Steward Healthc Epidemiol. 2023 Oct 25;3(1):e186. doi: 10.1017/ash.2023.467 (PMC10654958; doi:10.1017/ash.2023.467)
Supplement: Morelli et al. supplementary material [file S2732494X23004679sup001.docx]

**Table S1.** Breakdown of empiric MRSA and resistant gram-negative therapy

|  | Number with empiric therapy | Number with concordant positive cultures | Number with concordant definitive therapy |
| --- | --- | --- | --- |
| **Empiric anti-MRSA therapy** | 224 | 27 | 88 |
| Hospitalization within last 90 days with IV antibiotics | 61 | 8 | 28 |
| Culture with MRSA in past year | 18 | 7 | 16 |
| Either hospitalization within last 90 days/IV antibiotics OR culture with MRSA in past year OR IVDU OR ESRD with HD | 82 | 13 | 41 |
| **Empiric antibiotics to treat resistant gram-negative organisms** | 217 | 33 | 67 |
| Hospitalization within last 90 days with IV antibiotics | 60 | 14 | 24 |
| Culture with any resistant gram-negative organism (including both *P. aeruginosa* and other non-pseudomonal resistant gram-negative organisms in past year | 21 | 5 | 13 |
| Either hospitalization within last 90 days/IV antibiotics OR culture with any resistant gram-negative organism (including both *P. aeruginosa* and other non-pseudomonal resistant gram-negative organisms) in past year | 74 | 18 | 34 |

ESRD: end-stage renal disease, HD: hemodialysis, IVDU: intravenous drug use, MRSA: methicillin-resistant *Staphylococcus aureus.*
